# Evaluation of Metabolic Parameters on Use of Newer Antiepileptics Versus Conventional Antiepileptics in Patients of Generalised Tonic-Clonic Seizure: An Observational Study

**DOI:** 10.7759/cureus.35181

**Published:** 2023-02-19

**Authors:** Suhasini Dehury, Pallavi Patro, Lorika Sahu, Laxmipriya Nayak, Ashok k Mallik

**Affiliations:** 1 Pharmacology and Therapeutics, Srirama Chandra Bhanja (S C B) Medical College and Hospital, Cuttack, IND; 2 Pharmacology and Therapeutics, Sanjay Gandhi Post Graduate Institute of Medical Science, Lucknow, IND; 3 Pharmacology and Therapeutics, Bhima Bhoi Medical College and Hospital, Balangir, IND; 4 Neurology, Srirama Chandra Bhanja (S C B) Medical College and Hospital, Cuttack, IND

**Keywords:** levetiracetam, oxcarbamazepine, gtcs, newer antiepileptics, metabolic adrs

## Abstract

Background and objective

Epilepsy is the commonest serious neurological condition and around 50 million people live with epilepsy (PWE). Primary and secondary generalised tonic-clonic seizures (GTCS) together constitute up to 50% of adult and adolescent epilepsy. GTCS respond well to broad-spectrum AEDs like valproate, phenytoin, levetiracetam, lamotrigine, and topiramate. Carbamazepine and oxcarbazepine are considered alternatives. Metabolic derangements with the conventional AEDs (phenytoin causes loss of bone mass in women, phenytoin and carbamazepine produce increases in serum lipid and C-reactive protein, weight gain with valproate) are well documented. But, there is limited data regarding the effect of the newer AEDs on metabolic parameters. Thus, this study was undertaken to assess the effects of the newer AEDs on the metabolic profile of patients with epilepsy.

Material and methods

A prospective observational study was conducted in the Department of Pharmacology, in collaboration with the Department of Neurology at S.C.B. Medical College and Hospital, Cuttack. 100 diagnosed patients with GTCS receiving monotherapy of either conventional or newer anti-epileptics were included in the study. Their metabolic parameters like total cholesterol, serum sodium, serum TSH and fasting blood glucose were collected at baseline, three months, and six months. ADRs were collected during the entire study period and causality assessment was done using WHO-UMC Causality Assessment Scale. All the data were analysed using SPSS 20.0 after applying appropriate statistical tests.

Results

There was a significant increase in total cholesterol in all four groups (p=0.002) but a pathological increase in the phenytoin and oxcarbazepine groups. There was a significant rise in the serum TSH levels in all groups except levetiracetam, but a pathological increase was seen with phenytoin and valproate, i.e., the conventional ones. Statistically significant hyponatremia was seen with valproate and oxcarbazepine. A rise in the FBS was seen with both phenytoin and valproate (p=0.002) but a pathological rise was seen with phenytoin. Out of the total reported ADRs, 53.5% were seen with conventional AEDs, and the rest 46.5% were seen with newer ones.

Conclusion

The advent of newer anti-epileptic drugs has unfolded wider horizons to the treatment of epilepsy. Each of these drugs has a unique mechanism of action, making it less prone to resistance. Metabolic derangements are a key determinant in the compliance of these drugs as they can predispose to other co-morbidities. Periodic monitoring of the various metabolic parameters is useful and together with patient counselling can improve the effectiveness of the anti-epileptic drugs.

## Introduction

Epilepsy is one of the most common and serious neurological conditions, affecting 0.5%-1% of the population and accounting for a notable proportion of the world’s disease burden. Today, there are an estimated 50 million people with epilepsy (PWE) [1], 80% of whom live in developing countries. India contributes to one-sixth of the global burden of epilepsy [2]. Primary and secondary generalised tonic-clonic seizures (GTCS) constitute up to 50% of adult and adolescent epilepsy [3]. At any given point in time, 4-10 people per 1,000 in a general population suffer active epilepsy, i.e., continuing seizures or needing treatment [1]. About 70% of PWE become seizure-free with the appropriate use of medications [4]. Antiepileptic pharmacotherapy for adults can be put to an end after a seizure-free period of 2-5 years [5,6]. Antiepileptic drugs (AEDs) are a major treatment consideration for PWE, and therefore a primary concern in choosing the appropriate drug. GTCSs respond well to AEDs like valproate, phenytoin, levetiracetam, lamotrigine, and topiramate [3]. Carbamazepine and oxcarbazepine are considered alternatives.

Various treatment options have been prescribed for GTCSs, and adverse reactions related to AEDs are a major limiting factor. With the discovery of new AEDs, there has been a significant advancement in the treatment of epilepsy over the past decade. Newer AEDs offer the potential advantages of fewer drug interactions, unique mechanisms of action, and a broader spectrum of activity. They have also been used as an adjunctive treatment for refractory epilepsy [7]. Most new AEDs involve less teratogenicity, and they have a milder effect on the patient’s hormone secretion and bone and lipid metabolism [8].

Metabolic derangements with conventional AEDs are well documented. Phenytoin causes loss of bone mass in women; phenytoin and carbamazepine produce increases in serum lipid and C-reactive protein; and valproate causes weight gain [9]. But there is limited data regarding the effect of the newer AEDs on metabolic parameters. To address this research gap, this study was undertaken to assess the effects of the newer AEDs on the metabolic profile of patients with epilepsy.

## Materials and methods

This study was a prospective observational study conducted with the primary objective of evaluating the effect of the newer and conventional antiepileptics on metabolic parameters. The study was conducted over a period of two years from 2015 to 2017 in the Department of Pharmacology, in collaboration with the Department of Neurology, at S.C.B. Medical College and Hospital, Cuttack. Ethical clearance was provided by the Institutional Ethics Committee (IEC/IRB No.474) prior to the start of the study. Written, informed consent was established, and confidentiality was maintained throughout the study.

The study population consisted of diagnosed patients with GTCS attending the neurology Out-patient Department. Those receiving monotherapy of either conventional or newer antiepileptics were included in the study. The patients who denied consent for follow-up and those with psychiatric illnesses were excluded from the study. A total of 100 patients were observed during the study period. Their metabolic parameters, like total cholesterol, serum sodium, serum TSH, and fasting blood glucose were collected at baseline, three months, and six months. Each patient was followed up for a period of six months. Also, the patients were advised to report to the doctor in case of any adverse drug reaction or intolerance during the course of the treatment. The causality assessment was done using the WHO-UMC Causality Assessment Scale [10].

Drugs and doses

GTCS patients receiving Phenytoin at a dose of 100 mg thrice daily were under group-A, patients receiving Valproate at a dose of 20-40 mg/kg of body weight were under group B, patients receiving Levetiracetam at a dose of 1,500-2,000 mg in divided doses were under group-C, and patients receiving Oxcarbazepine at a dose of 150-300 mg twice daily were under group-D. Twenty-five patients in each group, i.e., a total of 100 patients with GTCS, were observed during the study period. For evaluation of metabolic ADRs, reports of the total cholesterol, thyroid stimulating hormone (TSH), serum sodium, and fasting blood sugar (FBS) levels were collected from each group and studied at baseline, three-month, and six-month intervals.

The patient details were collected on a preformed case record form and later entered into a Microsoft Excel spreadsheet. All the data were analysed using SPSS 20.0. Numerical data were expressed in terms of mean and standard deviations. Metabolic parameters were described using the median and interquartile range, and comparison was done using the Wilcoxon Signed Rank test. Any difference was considered statistically significant at p<0.05.

## Results

A total of 100 patients were evaluated. The mean age was 31 ± 14.1 years. The majority were males (60%) and rest were females (40%). Various adverse drug reactions (ADRs) were seen both with the conventional AEDs and newer AEDs (Table 1). The majority of ADRs (53.5%) were seen with conventional AEDs, and the rest (46.5%) were seen with the newer ones. CNS side-effects like impaired concentration, drowsiness, anxiety, depression, and sedation were the most common (38%), followed by metabolic side-effects.

**Table 1:** Demographic profile and types of ADRs

Age in years (mean±SD)	31 ± 14.1
Gender distribution [n=100]	
Males (%)	60 (60%)
Females (%)	40(40%)
Conventional AEDs	
total number used (n=50) ADR caused (n=98)	55 (56.1%)
Newer AEDs	
total number used (n=50) ADR caused (n=98)	43 (43.8%)
Types of ADRs (%)	
CNS	38%
Metabolic	25%
Dermatologic	20%
Gastrointestinal	22.5%
Genitourinary	5%
Musculocutaneous	5%
Constitutional	1.5%
Ophthalmic	1%
Causality assessment (n=100)	
Possible	60 (60%)
Probable/Likely	11.5 (11.5%)
Unlikely	28.5 (28.5%)

For the evaluation of metabolic ADRs, reports of the total cholesterol, thyroid stimulating hormone (TSH), serum sodium, and fasting blood sugar (FBS) levels were studied, and conventional AEDs (phenytoin, valproate) were compared to newer AEDs (levetiracetam, oxcarbazepine) at baseline, three months, and six months (Table 2).

**Table 2:** Metabolic parameters at baseline, three months, six months

Metabolic parameters	Time intervals	Phenytoin (n=25)	P-value*	Valproate (n=25)	P-value*	Levetiracetam (n=25)	P-value*	Oxcarbazepine (n=25)	P-value*
Total cholesterol (mg/dl) median (IQR)	Baseline	160 (148.5–166.3)		160 (148–164.5)		151 (138.8–165)		155.5 (150­–161.3)	
	3 months	174 (159–189)	0.002	168 (156.5–170.5)	0.002	159 (149– 176)	0.002	159 (150.5–167.5)	0.002
	6 months	213 (207–220)	0.002	172 (166.8–180)	0.002	162.5 (158–181.3)	0.002	210 (203.8– 211.3)	0.002
TSH(mIU/L) median (IQR)	Baseline	2.5 (2–3)		2 (1–3)		2 (1–3)		2 (2–3)	
	3 months	3 (2–4)	0.125	4 (2–4)	0.004	2 (1–3)	1	4 (2–5)	0.02
	6 months	7 (3–17.8)	0.02	6.5 (3–10.3)	0.002	2.5 (2–3)	0.06	5 (4–8.8)	0.002
Sr sodium (mEq/L) median (IQR)	Baseline	138.5 (137–140)		139.5 (138.5–142)		138 (136.8–139)		139 (135.8–141.3)	
	3 months	138 (136.8–139.5)	0.6	138 (137.3–131)	0.5	139 (137.8–140)	0.004	133 (130–137.5)	0.002
	6 months	137 (135.8–140.5)	0.2	130 (128–140.5)	0.002	140 (139– 141)	0.004	129.5 (125.8–136.3)	0.002
FBS (mg/dL) median (IQR)B	Baseline	94 (87.5–99.3)		89.5 (78.8–95.8)		94 (80.5–100.5)		90 (84.5– 97.8)	
	3 months	104 (93.8–109.3)	0.002	94 (88.8–101.3)	0.002	94.5 (80.5–109.3)	0.03	89 (87.3–102.3)	0.07
	6 months	113.5 (102–120.8)	0.002	102 (95.8–108)	0.002	93.5 (80.5–116.3)	0.06	92.5 (87.8–104.3)	0.05

Conventional drugs like phenytoin and valproate, along with newer AEDs like levetiracetam and oxcarbazepine, were considered for the evaluation of metabolic ADRs. A total of 100 patients were evaluated for any change in their in total cholesterol, TSH, serum sodium, and FBS. Twenty-five patients were given each of the drugs mentioned above. There was a significant increase in total cholesterol in all the four groups (p=0.002) and a pathological increase in the phenytoin and oxcarbazepine group. There was a significant rise in the serum TSH levels in all groups except levetiracetam, but pathological increase was seen with phenytoin and valproate, i.e., the conventional ones. Statistically significant hyponatremia was seen with valproate and oxcarbazepine. Rise in the FBS was seen with both phenytoin and valproate (p=0.002), but pathological rise was seen with phenytoin.

## Discussion

This study was designed to demonstrate the effects of phenytoin, valproate, levetiracetam, and oxcarbazepine on metabolic parameters such as total cholesterol, serum TSH, serum sodium, and FBS after three months and six months of treatment. The study showed an increase in total cholesterol with both the conventional and the newer antiepileptics, but a pathological rise was seen with phenytoin and oxcarbazepine. A total cholesterol level of <200mg/dl is considered to be desirable [11]. Previous studies have documented a rise in total cholesterol levels with phenytoin [12,13]. Mechanisms are poorly understood but may be caused by the activation of the pregnane X receptor (PXR), which is a major regulator of drug metabolism and drug-drug interactions contributing to hypercholesterolemia [14]. Similarly, there was a rise in serum total cholesterol, triglycerides, LDL-C/HDL-C apolipoprotein AI (ApoAI), and apolipoprotein B (ApoB) in children treated with sodium valproate [15]. On the contrary, a study by Kantoush et al. had varying results. In that study, children with epilepsy receiving valproate had lower total cholesterol (TC), low-density lipoprotein cholesterol (LDL-C), much lower-density lipoprotein cholesterol (VLDL-C) LDL-C/HDL-C ratio and a higher high-density lipoprotein Cholesterol (HDL-C) ratio than controls [16]. The mechanism behind the lipid profile changes due to valproic acid is still unclear. A possible mechanism may be insulin resistance and hyperinsulinemia, resulting in impaired lipid transport and lipogenesis [17]. Another possible mechanism may be the induction of cytochrome p (CYP) enzymes that play a role in metabolism and the biosynthesis of cholesterol. Studies have shown that there has been a significant rise in total cholesterol with oxcarbazepine, but levetiracetam showed no significant changes [18].

There was a significant increase in serum TSH levels in patients on valproate and phenytoin as compared to levetiracetam and oxcarbazepine. Similar results were seen in a study by Dahiya et al., where patients receiving phenytoin monotherapy developed hypothyroidism with a rise in TSH levels [19]. A study by Pattan et al. showed that with long-term phenytoin administration, there was a lowering of T4 level in the setting of normal TSH level [20]. Other studies showed that there was the development of subclinical hypothyroidism in children receiving AEDs, particularly valproate [21,22]. Thyroid hormones play an important role in the physiological process and a disturbance in them may lead to metabolic syndrome with multiple systems involved. Hence, it is important to keep watch on the hormone levels of patients on antiepileptics. According to the literature, it is most likely that AEDs affect hepatic microsomal enzyme systems and accelerate the metabolism of thyroid hormones. Human uridine diphosphate glucuronosyl transferase (UGT) could also be responsible for glucuronidation and thus metabolisation of thyroid hormones, as high levels of UGT have been observed after AED therapy. However, it was not established that valproate induced thyroid dysfunction. This could be due to enzyme induction.

In the present study, there was a significant pathological decrease in serum sodium in the valproate and oxcarbazepine group. These findings are similar to the studies by Patel et al. [23] and Branten et al. [24], which concluded that sodium valproate can cause hyponatremia with SIADH-like syndrome. Previous studies also showed hyponatremia with oxcarbazepine, presenting as nausea, fatigue, and worsening of GTCS [25,26]. Possible reasons could be a direct effect of oxcarbazepine on renal-collecting tubules or enhanced responsiveness to the circulating antidiuretic hormone (ADH).

There was a significant pathological increase in fasting blood glucose with phenytoin and valproate. A previous study by Rubeaan et al. showed that phenytoin-induced hyperglycemia could be primarily due to the inhibition of insulin release and increased insulin insensitivity [27]. In the present study, we found that hyperglycemia correlated with valproate. Similar results were found in a study performed on diabetic rabbits, where valproate resulted in hypoglycaemia [28]. In contrast, a study by Rakitins et al. showed that valproate was associated with weight gain, thus resulting in other metabolic changes and hyperinsulinemia [29].

## Conclusions

The advent of newer antiepileptic drugs has uncovered wider horizons for the treatment of epilepsy. Each of these drugs has a unique mechanism of action, making it less prone to resistance. Metabolic derangements are a key determinant for the compliance of these drugs, as they can result in a predisposition to other co-morbidities. For instance, a change in the lipid profile or blood sugar profile can put the epilepsy patient at risk of cardiovascular diseases or diabetes. Although there are studies indicating better side-effect profiles for the newer AEDs as compared to the conventional ones, further studies are needed to confirm their superiority. Periodic monitoring of the various metabolic parameters is useful, and, together with patient counselling, can improve the effectiveness of the antiepileptic drugs.
